# tRNA-derived small RNAs in digestive tract diseases: Progress and perspectives

**DOI:** 10.1016/j.gendis.2024.101326

**Published:** 2024-05-13

**Authors:** Mingrui Liu, Xiaojun Zhuang, Haiqing Zhang, Weidong Ji, Gang Yuan

**Affiliations:** a Center for Translational Medicine, The First Affiliated Hospital, Sun Yat-sen University Guangzhou, Guangdong 510080, China; b School of Pharmaceutical Sciences, Sun Yat-Sen University, Guangzhou, Guangdong 510006, China; c Department of Geriatrics, The First Affiliated Hospital, Sun Yat-Sen University, Guangzhou, Guangdong 510080, China; d Department of Gastroenterology, The First Affiliated Hospital, Sun Yat-sen University, Guangzhou, Guangdong 510080, China; e International Medical Center, The First Affiliated Hospital, Sun Yat-Sen University, Guangzhou, Guangdong 510080, China; f Phase I Clinical Trial Center, The First Affiliated Hospital, Sun Yat-Sen University, Guangzhou, Guangdong 510080, China

**Keywords:** Biogenesis, Biological function, Digestive tract diseases, Mechanism, tRNA-derived small RNAs (tsRNAs)

## Abstract

tRNA-derived small RNAs (tsRNAs) are non-coding small RNAs that are produced through the precise cleavage of tRNA molecules under specific conditions. tsRNA has multiple functions, including inhibiting translation, acting in association with classical small RNA effector mechanisms, or acting in conjunction with Argonaute proteins that affect cell proliferation, migration, cycle, and apoptosis. Recent studies have revealed the clinical potential of tsRNAs in numerous diseases. This article aims to provide a comprehensive and up-to-date review of the classification and biological function of tsRNAs in gastrointestinal diseases. Furthermore, this review explores the underlying mechanisms by which tsRNAs are believed to exert their effects in both tumor and non-tumor digestive tract diseases. Therefore, specific tsRNAs prove promising for disease diagnosis, prognosis prediction, and therapeutic interventions as novel biomarkers for digestive tract diseases.

## Introduction

tRNA-derived small RNAs (tsRNAs) are functional non-coding RNAs produced via precise cleavage of mature or pre-tRNAs under physiological or pathological conditions and are categorized as tRNA-derived fragments (tRFs) or tRNA halves (tiRNAs).^1^ Initially discovered in tumor cells in the late 1970s, tsRNAs were first considered random degradation products of tRNA with an unknown specific role.^2^ However, recent studies have begun to elucidate the roles of tRNA, with a particular focus on tsRNAs in viruses,^3^ plants,^4^ parasites,^5^ and various human diseases.^6^ Differential expression of tsRNA has been observed in multiple species, is highly conserved, and exhibits biological functions such as regulating translation, participating in epigenetics, and interacting with proteins. Over the past decade, researchers have recognized tsRNAs as important molecules involved in diverse cellular processes and reported that their expression and modification exhibit tissue and cell specificity. Moreover, strong associations between tsRNAs and various diseases have been reported.^7^ tsRNAs have been shown to play a role in the pathogenesis of various diseases, impacting immunity, metabolism, and malignant tumor development,^8^ which suggests that tsRNAs hold potential for emerging clinical applications.

Emerging evidence suggests that tsRNAs play vital roles in the occurrence and development of digestive tract diseases. Studies analyzing the sequencing results of clinical samples from gastrointestinal diseases have demonstrated that changes in the expression profile of tsRNAs can be used for disease diagnosis and prognosis prediction. Furthermore, researchers have achieved significant therapeutic effects by utilizing targeted tsRNA agents in animal models. However, existing reviews have primarily focused on tsRNA research in the context of tumors, and there is a lack of comprehensive reviews exploring the application of tsRNA in non-tumor digestive tract diseases. This review aims to address this gap by examining the molecular mechanisms and clinical value of tsRNAs in digestive tract diseases, including both digestive tract tumors and non-tumor digestive tract diseases. A systematic overview of the correlation between tsRNAs and digestive tract diseases is expected to support the development of new therapeutic options based on tsRNAs for digestive tract diseases.

### Biogenesis and classification of tsRNA

**Figure 1:**
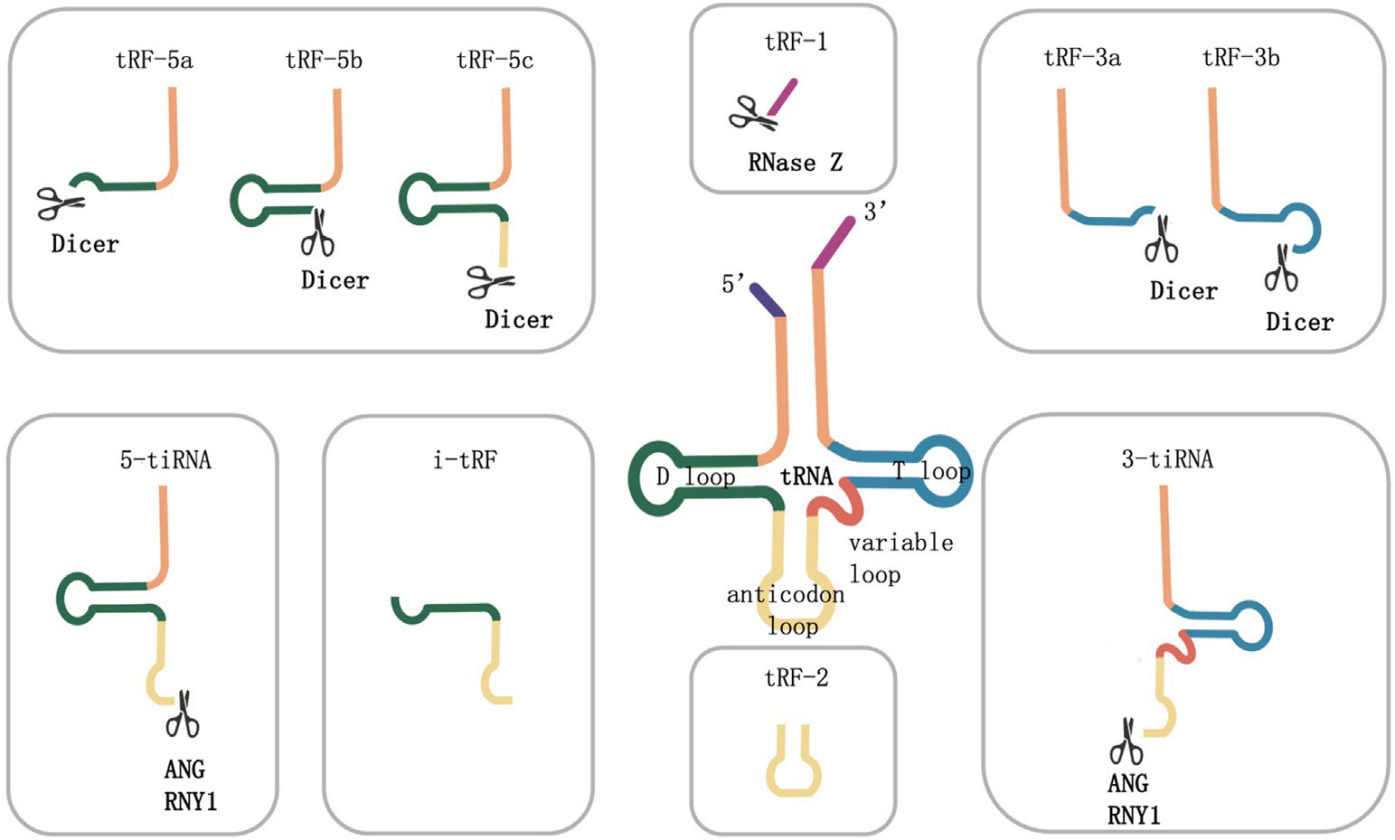
Biogenesis and classification of tsRNAs. tsRNA is divided into two types: tRFs and tiRNAs. tRFs include tRF-1, tRF-2, tRF-3, tRF-5, and i-tRF. tRF-1 is produced by cleavage of the precursor tRNA molecule at the 3′ end by endonuclease Z (RNase Z). tRF-2 includes the anticodon stem and ring regions of tRNA. tRF-3a and tRF-3b are produced by cleavage of the 3′ end T ring of mature tRNA. tRF-5a, tRF-5b, and tRF-5c originate from cleavage at different positions from the 5′ end to the D ring of mature tRNA. i-tRFs mainly originate from the internal regions of mature tRNA.

The process of tsRNA biogenesis is illustrated in Figure 1, tsRNAs are formed through the degradation of tRNA by various nucleases, resulting in the generation of two distinct molecules: tRFs and tiRNAs.^9^ tRFs are tRNA fragments that typically range in length from 14 to 30 nucleotides (nt),^6^^,^^10^ and they are produced via specific cleavage events that can occur either during the maturation of tRNA or from precursor tRNA transcripts.^11^ Under specific stress conditions, angiopoietin (ANG) specifically cleaves tRNA into two halves, known as tiRNAs.^12^^,^^13^ These tiRNAs consist of a 5′-tRNA half and a 3′-tRNA half, with lengths of approximately 29–50 nt.^13^

tRFs are classified according to the specific regions of precursor or mature tRNA where they are cleaved.^14^ Recent research has identified five categories of tRFs: tRF-1, tRF-2, tRF-3, tRF-5, and i-tRF.^10^ The processing of tRFs involves nuclear lysis and nucleic acid exonucleases.^15^ tRF-1, also known as 3′U-tRFs, is derived from the uracil-rich region at the 3′ terminus of the precursor tRNA molecule, and this sequence is generated when the endonuclease Z (RNase Z) removes the 3′-tail sequence during tRNA maturation.^16^ A new class of tRFs called tRF-2 has been discovered in breast cancer cells and includes the tRNA anticodon stem and loop regions. These tRFs are derived from tRNA^Glu^, tRNA^Asp^, tRNA^Gly^, and tRNA^Tyr^.^17^^,^^18^ tRF-3 is produced by cleaving the 3′ end T-loop of mature tRNA and contains transcribed CCA trinucleotides, which are approximately 18–22 nt in length and can be further divided into tRF-3a and tRF-3b.^19^ tRF-5 originates from the 5′ end of mature tRNA, where cleavage of the D-loop results in transcripts ranging from 1 to 30 bases in length. Depending on the number of nucleotides, tRF-5 can be further divided into tRF-5a, tRF-5b, and tRF-5c.^19^ The processing of a few specific tRF-3 and tRF-5 variants is dependent on the Dicer enzyme.^20^ Finally, i-tRFs mainly originate from the internal regions of mature tRNA.^10^

tiRNAs, also known as tRNA-derived stress-induced RNAs, typically range in length from 28 to 36 nt. Most tiRNAs are generated by various stress stimuli such as heat shock, cold shock, phosphate starvation, hypoxia, and oxidation,^13^ although a few tiRNAs are present in normal cells. tiRNAs are produced through the action of ribonuclease ANG and Ro60-related Y1 (RNY1), which cleave mature tRNA at different positions.^21^ The cleavage site at the 5′ end of the anticodon loop of mature tRNA is referred to as 5′tiRNA, and the cleavage site at the 3′ end of the anticodon stem of mature tRNA is called 3′tiRNA.^22^ Furthermore, tiRNAs exhibit specific methylation and terminal modifications, including cyclic phosphorylation, 5-OH modification, and aminoacyl modification.^23^

### Biological functions of tsRNAs

**Figure 2:**
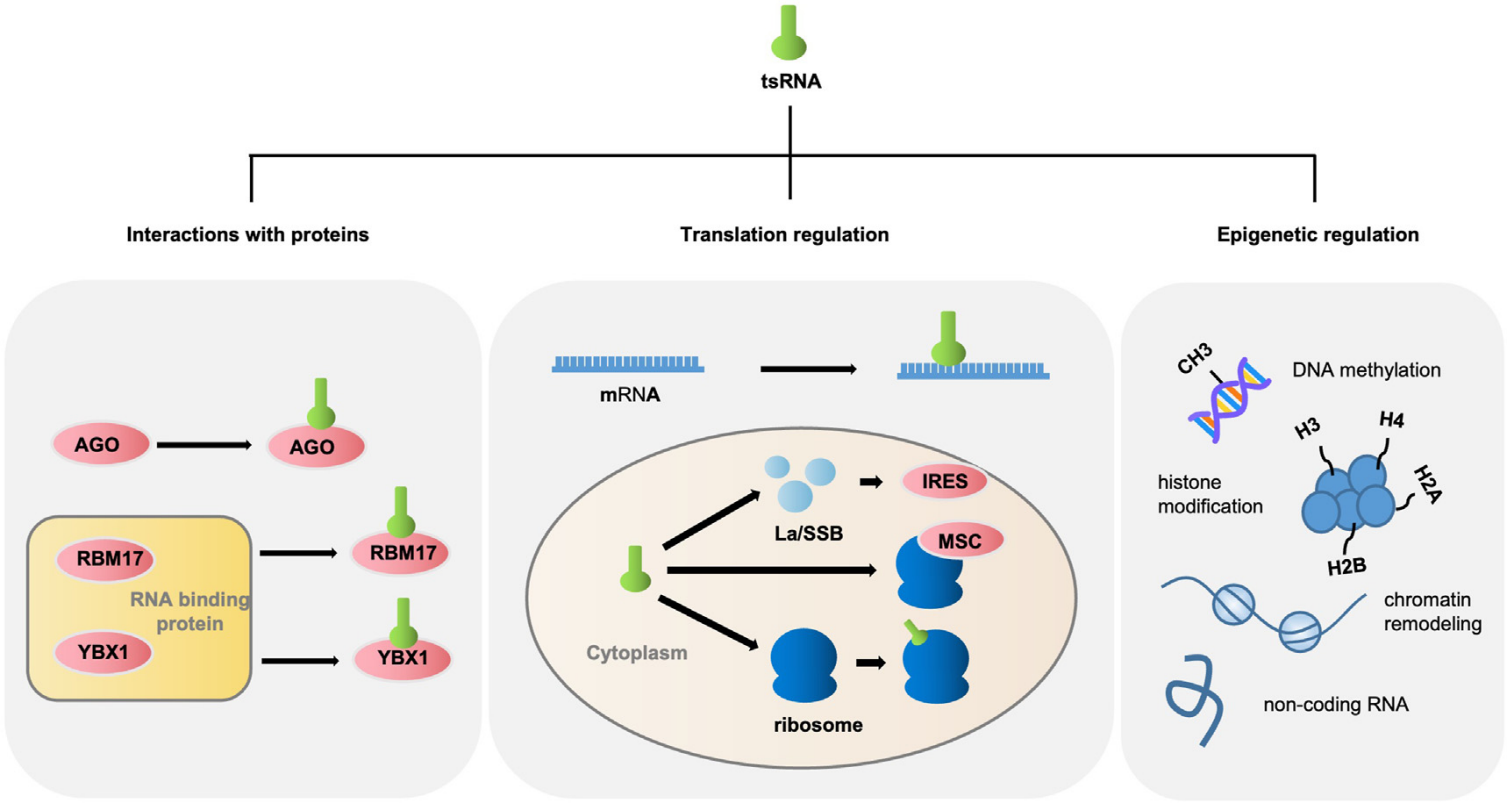
Biological functions of tsRNAs. The interaction between tsRNA and RNA binding proteins such as AGO (Argonaute) protein, RBM17, and YBX1 mediates gene silencing; TsRNA can regulate translation through the classic microribonucleic acid-like pathway and promote ribosomal biogenesis; TsRNA participates in various epigenetic processes such as DNA methylation, histone modification, chromatin remodeling, and regulation of non-coding RNA (ncRNA). RBM17, RNA binding motif protein 17; YBX1, Y-box binding protein 1.

tsRNAs are known to exhibit multiple functions. Certain tsRNAs can inhibit translation, while others have been implicated in the classical small RNA effect mechanism. Others have been reported to bind to Argonaute (AGO) proteins.^24^ This section details the potential mechanisms of tsRNAs reported in the literature, which are summarized in Figure 2.

### Interactions with proteins

Several studies have emphasized the role of AGO proteins in gene silencing mediated by tsRNA. AGO proteins, including different subtypes such as AGO1, AGO2, AGO3, and AGO4, could affect the RNA silencing pathway as core components of the RNA-induced silencing complex (RISC).^25^ Moreover, the relationships between tRFs and AGO protein subtypes can vary. For instance, tRF-3 and tRF-3003a, derived from the 3′ end of tRNA-Cys^GCA^, induce the silence of a target gene called Janus kinase 3 (JAK3) in an AGO2-dependent manner.^26^ Moreover, tRF-3 derived from tRNAlys3 could bind with AGO2 and regulate gene expression via RNA interference in HIV-1-infected cells.^27^ Additionally, Zhong et al reported that glycine tRNA fragments (Gly-tRFs) generated from the tRNA precursor could selectively interact with AGO3, which could mediate the down-regulated expression of Sirt1 to trigger alcohol-induced liver injury and steatosis.^28^ Human tsRNA and AGO1 have both been shown to be involved in the processing of itRFs in planarian,^29^ and tsRNA in drosophila is closely related to the mediation of AGO1 and AGO2.^30^^,^^31^ Overall, interactions between tsRNA and different AGO protein subtypes have been highlighted in regulating gene expression in various biological contexts. Several investigations have revealed that tsRNA possesses the capability to engage in gene suppression via interaction with other RNA-binding proteins. For example, Han et al discovered that a specific type of tRF, namely tiRNA-Gly, can directly bind to RBM17 (RNA binding motif protein 17), thereby exerting carcinogenic influences in papillary thyroid cancer.^32^ Furthermore, other researchers observed that tRFs generated under stress stimulation impede the stability of carcinogenesis-associated transcripts in breast cancer cells by substituting the 3′ untranslated region within the RNA binding protein YBX1 (Y-box binding protein 1).^18^ A study on the differentiation of mouse embryonic stem cells observed that up-regulated 5′-tsRNA can preferentially interact with the RNA binding protein Igf2bp1 (insulin-like growth factor 2 mRNA-binding protein 1), affecting the stability of transcripts.^33^

### Translation regulation

tsRNA can be translated and regulated through the classic microRNA maturation pathway. Both tiRNAs and tRFs can inhibit translation via distinct mechanisms. Yamasaki et al transfected the 5′-tiRNAs into the human osteoblast cell line U2OS and observed their role in translational inhibition independent of phospho-eIF2alpha.^22^ The translation inhibition by 5′-tiRNAs necessitates a minimum of four guanosine residues at the 5′ terminal, whereas tRF-5 merely requires two guanosine residues at the 3′ terminal.^34^ tRFs have been shown to participate in regulating mRNA stability and translation via microRNA-like effects.^35^ For example, Ivanov et al have revealed that 5′-tsRNAs can directly interact with target mRNA through sequence complementarity or indirectly interact with specific RNA binding proteins at multiple stages, thereby modulating the role of retinoic acid in the differentiation of mouse embryonic stem cells.^36^ In cases where mRNA lacks target sites, a specific Val-tRF could directly inhibit translation by limiting peptide bond formation.^37^ However, Sobala and Hutvagner reported that the inhibition of protein translation by 5′tRFs is unrelated to mRNA cyclization.^34^ Certain tsRNAs can govern the process of protein translation by facilitating ribosome biogenesis.^35^ The RNA chaperone La/SSB (lupus-associated antigen with the HUGO gene name of Sjögren syndrome B) can bind to the stem-loop IV within the internal ribosome entry site of the hepatitis C virus, and tRF_U3_1 can isolate a limited number of La/SSB in the cytoplasm to negatively regulate internal ribosome entry site-mediated translation initiation.^38^ For other organisms, diverse ways in which tRFs can interact with ribosomes to regulate protein translation have been reported. In arabidopsis, tRNA^Ala^ (AGC) and tRNA^Asn^ (GUU) were observed to be involved in multi-ribosome interactions and act as a general regulator of the plant translation process to inhibit protein synthesis.^39^ In yeast, the tRFs from ribosome-associated non-coding RNAs were observed to bind to the ribosome *in vitro*, resulting in the regulation of protein biosynthesis.^40^ In halophilic archaea halophytes, Val-tRF bound directly to the ribosome and interfered with the peptidyl transferase activity, leading to a reduction in protein synthesis.^37^ In *Trypanosoma brucei*, the tRNA^Thr^ 3′ half was observed to bind to the ribosome and further stimulate translation.^41^

### Epigenetic regulation

Recently, tsRNAs have been investigated for potential involvement in the modulation of gene expression through diverse epigenetic processes, encompassing DNA methylation,^42^^,^^43^ histone modification,^44^ chromatin remodeling,^45^ and non-coding RNA regulation.^46^ Among potential pathways, tsRNAs may inhibit the activity of repetitive sequences, such as transposable elements, by affecting Setdb1 (SET domain bifurcated histone lysine methyltransferase 1)-mediated trimethylation of histone H3K9 or Dnmt1 (DNA methyltransferase 1)-mediated DNA methylation, thereby inhibiting most long-terminal repeat retrotransposons.^47^ Moreover, Schorn et al discovered that 3′CCA tRFs can target and inhibit endogenous retroviral activity by binding to the highly conserved primer binding site of a long-terminal repeat retrotransposon in mice.^43^ Another potential pathway is involvement in non-coding RNA regulation. Durdevic et al revealed that tRNA fragments can inhibit the activity of Dicer 2 on long double-stranded RNAs, leading to enriched double-stranded RNAs and insufficient small interfering RNAs in drosophila.^46^ Finally, tRFs can participate in the accessibility of chromatin. Boskovic et al reported that tRF-5′tRF-Gly-GCC (tRF-GG) plays a role in regulating various non-coding RNAs by specially targeting U7 small nucleolar RNA involved in histone supply and heterochromatin-mediated transcriptional inhibition.^45^

### Role and mechanism of tsRNAs in digestive tract diseases

The potential role of tsRNAs in digestive tract diseases has generated great interest, and numerous studies have demonstrated that tsRNAs hold significant clinical significance as possible diagnostic markers.^48^, ^49^, ^50^, ^51^, ^52^, ^53^, ^54^, ^55^, ^56^, ^57^, ^58^, ^59^, ^60^, ^61^, ^62^ For example, TRF-29-R9J8909NF5JP,^48^ tRF-19-3L7L73JD,^49^ tRF-33-P4R8YP9LON4VDP,^50^ tRF-23-Q99P9P9NDD,^51^ tRF-33-P4R8YP9LON4VDP,^50^ tRF-27-FDXXE6XRK45,^52^ tiRNA-5034-GluTTC-2,^53^ tRF-25,^54^ tRF-38,^54^ tRF-18,^54^ tRF-27-87R8WP9N1E5,^55^ tRF-31-U5YKFN8DYDZDD,^56^ and tRF-5026a^57^ have been designated as diagnostic markers for gastric cancer (GC). 5′-tiRNA-Val^58^ and 5′-tRF-GlyGCC^59^ have been reported as diagnostic markers for colorectal cancer (CRC). tRNA-ValTAC-3,^60^ tRNA-GlyTCC-5,^60^ tRNA-ValAAC-5,^60^ and tRNA-GluCTC-5^60^ are effective diagnostic markers for hepatocellular carcinoma (HCC). tRF-Val-CAC-005,^61^ tiRNA-His-GTG-001,^61^ and tRF-Ala-CGC-006^61^ can be used to diagnose non-alcoholic fatty liver disease (NAFLD). tsRNA-ValTAC-41,^62^ tsRNA-MetCAT-37,^62^ and tsRNA-ThrTGT-23^62^ can be used to diagnose pancreatic ductal carcinoma (PDAC). In addition, certain tsRNAs might be closely associated with disease incidence rates, while others exhibit potential for predicting disease prognosis and monitoring disease progression.^50^^,^^54^^,^^56^^,^^63^, ^64^, ^65^, ^66^, ^67^

**Figure 3:**
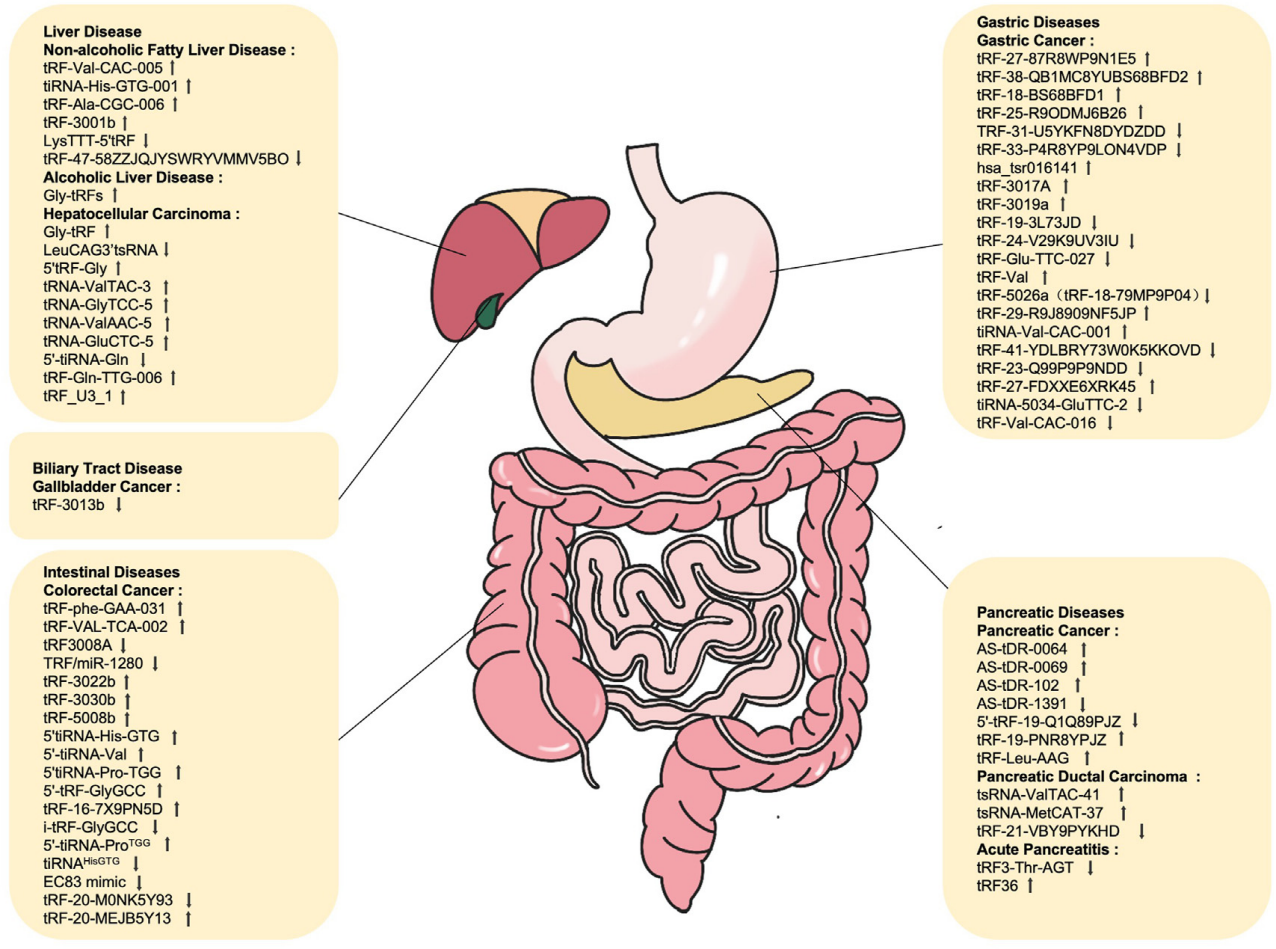
Expression of tsRNAs in digestive tract diseases.

Thus far, tsRNA has primarily been applied to the diagnosis and prognosis of gastrointestinal tumors. TRF-31-U5YKFN8DYDZDD^56^ and hsa_Tsr016141^67^ are considered predictive indicators of poor prognosis in GC. i-tRF-GlyGCC,^63^ 5′-tiRNA-Pro^TGG^,^64^ tRF-22-WB86Q3P92,^65^ tRF-22-WE8SPOX52,^65^ tRF-22-WE8S68L52,^65^ and tRF-18-8R1546D2^65^ are predictive indicators of poor prognosis in CRC. tRF-21-VBY9PYKHD^66^ is a predictive indicator of poor prognosis in PDAC. Numerous studies have identified target genes^68^, ^69^, ^70^, ^71^, ^72^, ^73^, ^74^, ^75^, ^76^, ^77^, ^78^, ^79^, ^80^, ^81^ and signaling pathways^57^^,^^72^^,^^75^^,^^82^, ^83^, ^84^, ^85^, ^86^, ^87^ of these tsRNAs. By further investigating the underlying mechanism of tsRNAs involved in the occurrence and development of diseases, novel treatment approaches are expected to be achieved. Figure 3 depicts the expression of tsRNAs in digestive tract diseases, and the molecular mechanisms of tsRNAs are summarized in Table 1.

**Table 1:** Mechanisms of tsRNAs in digestive tract diseases in the literature.

tsRNA	Disease	Trials	Mechanism	Reference
Protein interactions				
tRF-3017A	GC	*In vitro*	Silencing tumor suppressor factor NELL2 by binding to AGO protein	88
tRF-3019a	GC	*In vitro*	Directly regulating tumor suppressor gene FBXO47	71
tRF-19-3L7L73JD	GC	*In vitro*	Blocking G0/G1 phase cells	49
tRF-Val	GC	*In vitro*	Inhibition of downstream p53 pathways	68
tRF-Val	GC	*In vitro* and *in* vivo	Binding with EEF1A1 and promoting its interaction with MDM2	68
tiRNA-Val-CAC-001	GC	*In vitro*	Through Wnt/β- Catenin signaling targeting LRP6	69
tRF-41-YDLBRY73W0K5KKOVD	GC	*In vitro*	Targeting PAPSS2	70
tRF-Val-CAC-016	GC	*In vitro*	Regulating MAPK signaling pathway mediated by CACNA1d	83
tiRNA-Tyr-GTA	CRC	Bioinformatic prediction	Peroxisome proliferator-activated receptor (PPAR) signaling pathway	89
TRF/miR-1280	CRC	*In vitro*	Directly acting on Notch ligand JAG2	85
TRF3008A	CRC	*In vitro*	Weakening the stability of endogenous carcinogenic transcript FOXK1 by binding to AGO protein	91
tRF-3022b	CRC	*In vitro*	Regulating M2 macrophage polarization by binding to LGALS1 and MIF	92
5′iRNA-His-GTG	CRC	*In vitro*	Targeting LATS2 to regulate HIF1 α/angiopoietin axis	72
tRF-16-7X9PN5D	CRC	*In vitro*	Regulating eIF4E phosphorylation through MKNK1	73
tRF-Gln-CTG-026	Liver injury	*In vitro*	Inhibiting global protein synthesis by weakening the association between TSR1 and first 40S ribosomes	97
5′tsRNA-Gly-GCC	High-fat diet model	*In vivo*	Regulating Sirt6–FoxO1 pathway	86
Gly-tRFs	ALD	*In vitro and vivo*	Regulated by CYP2E1, acting on the Gly tRF/AGO3/Sirt1 axis	28
tRF-3001b	NAFLD	*In vitro and vivo*	Inhibiting the expression of autophagy related gene Prkaa1	76
Gly-tRF	HCC	*In vitro*	Targeting NDFIP2	75
5′-tRF-Gly	HCC	*In vitro*	Targeting CEACAM1	59
tRF_U3_1	HCC	*In vitro*	Chelating La/SSB	38
tRF-3013b	GBC	*In vitro*	Combining with AGO3	78
5′-tRF-19-Q1Q89PJZ	PDAC	*In vitro*	Regulating HK1 mediated glycolysis	79
tRF-19-PNR8YPJZ	PDAC	*In vitro*	Targeting downregulation of AXIN2 to activate Wnt pathway	80
tRF-Leu-AAG	PDAC	*In vitro*	Targeting UPF1	103
tRF36	AP	*In vitro*	Binding with IGF2BP3 to enhance RNA stability	81
Translation regulation				
LeuCAG3′tsRNA	HCC	*In vitro*	Binding to mRNA of ribosomal proteins (RPS28 and RPS15)	102
5′-tiRNA-Gln	HCC	*In vitro*	Interacting with EIF4A1	101
LysTTT-5′tRF	NAFLD	*In vitro and vivo*	Elevating the mRNA level of metabolic regulatory factors β-Klotho	98
Epigenetic regulation				
tRF-20-M0NK5Y93	CRC	*In vitro*	Combining MALAT1 to regulate selective splicing of SMC1A by SRSF2	74
tRF-21-VBY9PYKHD	PDAC	*In vitro and vivo*	Regulating AKT2/1-mediated heterogeneous nuclear ribonucleoprotein L (hnRNP L) phosphorylation	66
Acting on signaling pathways				
tRF-Glu-TTC-027	GC	*In vitro*	Mediating MAPK signaling pathway	82
tRF-24-V29K9UV3IU	GC	*In vitro*	Regulating Wnt signaling pathway	84
tRF-5026a	GC	*In vitro*	Acting on PTEN/PI3K/AKT signaling pathway	57

Notes: GC, gastric cancer; CRC, colorectal cancer; NAFLD, non-alcoholic fatty liver disease; HCC, hepatocellular carcinoma; PDAC, pancreatic ductal carcinoma; MAPK, mitogen-activated protein kinase; PTEN, phosphatase and tensin homologue; PI3K, phosphoinositide 3-kinase; AKT, protein kinase B; MALAT1, metastasis associated in lung adenocarcinoma transcript 1; SRSF2, serine and arginine rich splicing factor 2; JAG2, Jagged-2; FOXK1, Forkhead box class K1; MKNK1, MAPK interacting serine/threonine kinase 1; PAPSS2, 3'-phosphoadenosine 5'-phosphosulfate synthase 2; PPAR, peroxisome proliferator-activated receptor; LRP6, low-density lipoprotein receptor-related protein 6; NELL2, neural EGFL-like 2; HIF1α, hypoxia-inducible factor-1α; FBXO47, F-box protein 47; LGALS1, galactose agglutinin 1; MIF, macrophage migration inhibitory factor; SMC1A, structural maintenance of chromosomes 1A; hnRNP L, heterogeneous nuclear ribonucleoprotein L; TSR1, pre-rRNA-processing protein TSR1 homolog; Sirt6, sirtuin 6; FoxO1, Forkhead box protein O1; CYP2E1, cytochrome P450 2E1; AGO, Argonaute; AGO3, Argonaute 3; Prkaa1, protein kinase AMP-activated catalytic subunit alpha 1; La/SSB, lupus-associated (La) antigen with the HUGO gene name of Sjögren syndrome B; EIF4A1, eukaryotic translation initiation factor 4A1; NDFIP2, Nedd4 family interacting protein 2; CEACAM1, carcinoembryonic antigen-related cell adhesion molecule 1; HK1, hexokinase 1; AXIN2, axin 2; UPF1, Up-frameshift protein 1; IGF2BP3, insulin like growth factor 2 mRNA binding protein 3.

### Gastric disease

The development of tsRNA sequencing technology has allowed researchers to identify the expression levels of specific tsRNAs and tiRNAs in clinical GC samples. Yu et al reported that the levels of tRF-27-87R8WP9N1E5 in plasma were significantly higher in patients with GC as compared with those in healthy individuals.^55^ Zhu et al reported that patients with lower expression of tiRNA-5034-GluTTC-2 had significantly lower overall survival rates than those with higher expression.^53^ Additionally, Lin et al identified that several tsRNAs, including tRF-38-QB1MC8YUBS68BFD2, tRF-18-BS68BFD1, and tRF-25-R9ODMJ6B26, were significantly overexpressed in the plasma exosomes of patients with GC.^54^ Li et al reported that the expression level of tRF-29-R9J8909NF5JP was significantly increased in patients with GC.^48^ Li et al reported that tRF-27-FDXXE6XRK45 can be used to distinguish patients with GC, patients with gastritis, and healthy individuals.^52^ Zhang et al reported that tRF-23-Q99P9NDD in serum can be used to identify patients with GC and monitor their postoperative condition.^51^ These tsRNAs can be used as diagnostic markers. Furthermore, tsRNAs such as TRF-31-U5YKFN8DYDZDD and hsa_tsr016141 may be implicated as predictive indicators for poor prognosis in GC.^56^^,^^67^

Mechanistically, specific tsRNAs affect the pathogenesis of GC by regulating the proliferation, apoptosis, migration, and invasion of GC cells. tRF-33-P4R8YP9LON4VDP and a few tsRNAs inhibit GC cell proliferation and can serve as new diagnostic biomarkers and therapeutic targets for cancer.^50^ Wang et al reported that tRF-41-YDLBRY73W0K5KKOVD inhibits GC progression by targeting PAPSS2 (3′-phosphoadenosine 5′-phosphosulfate synthase 2).^70^ Tong et al discovered that up-regulated tRF-3017A in GC could silence the tumor suppressor factor NELL2 (neural EGFL-like 2) by binding to the AGO protein, thereby promoting the invasion and migration of cancer cells.^88^ Additionally, Zhang et al demonstrated that overexpressed tRF-3019a directly regulates the tumor suppressor gene FBXO47 (F-box protein 47) to enhance the proliferation, migration, and invasion of GC cells.^71^ Furthermore, Shen et al reported that enriched tRF-19-3L7L73JD blocks G0/G1 phase cells to inhibit GC cell proliferation and migration while inducing cell apoptosis.^49^ Similarly, tRF-24-V29K9UV3IU was observed to promote cell apoptosis by regulating the Wnt signaling pathway.^84^ In addition to these findings, other signaling pathways involved in GC have been reported. For example, down-regulated tRF-Glu-TC-027 mediates the MAPK (mitogen-activated protein kinase) signaling pathway,^82^ up-regulated tRF-Val inhibits the downstream pathway of p53,^68^ and tRF-5026a affects the PTEN (phosphatase and tensin homologue)/PI3K (phosphoinositide 3-kinase)/AKT (protein kinase B) signaling pathway.^57^ Additionally, tRF-Val-CAC-016 regulates the MAPK signaling pathway mediated by CACNA1d,^83^ while tiRNA-Val-CAC-001 is mediated by the Wnt/β-catenin signaling pathway targeting LRP6 (low-density lipoprotein receptor-related protein 6).^69^ These findings provide insight into the specific mechanisms by which tsRNA contributes to the pathogenesis of GC, and understanding these mechanisms may help in the development of targeted therapies and diagnostic approaches for GC.

### Colorectal disease

Researchers have reported that the abundance of certain tsRNAs is significantly altered in patients with CRC as compared with healthy individuals. For example, Wang et al conducted sequencing studies on CRC samples and observed 60 up-regulated and 48 down-regulated tRNAs in CRC,^89^ which suggests that tsRNA dysregulation may contribute to CRC development. Specific tsRNAs with clinical significance were identified. Up-regulation of tRF-phe-GAA-031 and tRF-VAL-TCA-002 in tumor tissue^90^ and down-regulation of i-tRF-GlyGCC levels^63^ are associated with shorter survival in patients with CRC. Overexpression of 5′-tiRNA-Pro^TGG^ can serve as an adverse prognostic factor for predicting short-term recurrence in patients with CRC.^64^ Additionally, a correlation between reduced expression of tRF3008A^91^ and high expression of 5′-tiRNA-Val^58^ and advanced/metastatic cancer in CRC came to light. Regarding these differentially expressed tsRNAs, Zhu et al established a diagnostic model based on tRF-22-WB86Q3P92, tRF-22-WE8SPOX52, tRF22-WE8S68L52, and tRF-18-8R1546D2 to assess prognostic risk.^65^ Relevant mechanisms by which tsRNAs contribute to the CRC are still being investigated. Lu et al reported that tRF-3022b binds to LGALS1 (galactose agglutinin 1) and MIF (macrophage migration inhibitory factor) in CRC cells, reducing M2 macrophage polarization by regulating MIF in M2 macrophages, thereby affecting CRC tumor growth.^92^ The carcinogenic effect of 5′iRNA-His-GTG in CRC, discovered by Tao et al, is related to its response to the hypoxic tumor microenvironment and is mediated by the HIF1α (hypoxia-inducible factor-1α)/ANG (angiopoietin) axis.^72^ Based on bioinformatics prediction, tiRNA-Tyr-GTA targets PPAR (peroxisome proliferator-activated receptor) signaling pathways to negatively regulate the apoptosis of epithelial cells, and tiRNA-Val-CAC plays a part in the response of cells to monoamine stimuli and inflammatory bowel disease.^89^ Luan et al validated that up-regulation of tRF-20-MEJB5Y13 by Dicer1 leads to hypoxia-induced CRC cell invasion and migration.^93^ The team reported that tRF-20-M0NK5Y93 regulates selective splicing of SMC1A (structural maintenance of chromosomes 1A) and inhibits colon cancer metastasis through the interaction of MALAT1 (metastasis associated in lung adenocarcinoma transcript 1) and SRSF2 (serine/arginine-rich splicing factor 2).^74^ In addition, tRF/miR-1280 directly acts on the Notch ligand JAG2 (Jagged-2) to suppress the growth and metastasis of CRC cells.^85^ tRF3008A binds to the AGO protein to weaken the stability of the endogenous carcinogenic transcript FOXK1 (Forkhead box class K1), thereby inhibiting the proliferation and migration of CRC cells.^91^ TRF-16-7X9PN5D can regulate eIF4E phosphorylation through MKNK1 (MAPK interacting serine/threonine kinase 1), thus playing an important regulatory role in the radiation resistance of CRC cells.^73^ Finally, differential tRFs may target corresponding messenger RNA to regulate the vitamin metabolism pathway and the cyclic guanosine monophosphate (cGMP)-protein kinase G (PKG) signaling pathway.^94^ Yang et al utilized the compound quercetin to regulate the expression of tiRNA^HisGTG^ and enhance the sensitivity of 5-Fluorouracil to CRC.^95^ The application of a 5′-tRF EC83 mimic derived from the gut microbiota demonstrated anti-tumor activity.^96^ Overall, the dysregulation of tsRNAs in CRC and their association with patient outcomes and cancer-related factors highlight their potential as diagnostic and prognostic biomarkers and their involvement in the molecular mechanisms underlying the development and progression of this disease. Mimics or inhibitors of tsRNA have the potential to become effective and selective therapeutic molecules.

### Liver disease

In liver diseases, tsRNAs can affect fat metabolism, protein synthesis, and sugar metabolism. Ying et al reported that tRF-Gln-CTG-026 inhibits global protein synthesis by weakening the association between TSR1 (pre-rRNA-processing protein TSR1 homolog) and pre-40S ribosome, thereby improving liver injury.^97^ In addition, 5′tsRNA-Gly-GCC can regulate the Sirt6 (sirtuin 6)-FoxO1 (Forkhead box protein O1) pathway to promote liver gluconeogenesis and may even act as a paternal epigenetic factor, mediating intergenerational inheritance of diet-induced metabolic changes.^86^ Researchers have observed that tsRNAs possess regulatory properties in various liver diseases, such as NAFLD, alcoholic liver disease, and HCC. In the case of alcoholic liver disease, Zhong et al reported that Gly-tRFs are regulated by CYP2E1 (cytochrome P450 2E1) in animal models, and the Gly-tRFs/AGO3/Sirt1 (sirtuin 1) axis may ultimately influence chronic liver steatosis.^28^ For NAFLD, differences in the expression profile of tsRNAs were observed in a cohort of 156 patients, and tRF-Val-CAC-005, tiRNA-His-GTG-001, and tRF-Ala-CGC-006 were significantly increased.^61^ In addition, a diet-induced NAFLD mouse model showed decreased LysTTTT-5′tRF^98^ and enriched tRF-3001b, whereby tRF-3001b inhibits the expression of the autophagy-related gene Prkaa1 (protein kinase AMP-activated catalytic subunit alpha 1) to affect triglyceride and cholesterol levels.^76^ TRF-47-58ZZJQJYSWRYVMMV5BO-mediated autophagy and cell apoptosis have significant effects on lipid damage and deposition *in vivo*.^99^ In HCC, Cho et al identified the liver cancer cell line Huh7 and obtained tRF_U3_1, which regulates viral gene expression by chelating La/SSB.^38^ Zhu et al identified elevated expression levels of tRNA-ValTAC-3, tRNA-GlyTCC-5, tRNA-Val-AAC-5, and tRNA-GluCTC-5 in the plasma exosomes of patients and reported their potential as new biomarkers.^60^ TRF-Gln-TTG-006 has been observed to distinguish HCC cases and healthy subjects with high sensitivity and specificity.^100^ In addition, the interaction between 5′-tiRNA Gln and EIF4A1 (eukaryotic translation initiation factor 4A1) inhibits translation and HCC progression.^101^ Gly-tRF targets NDFIP2 (Nedd4 family interacting protein 2) to promote tumor cell migration,^75^ LeuCAG3′tsRNA binds to the mRNA of ribosomal proteins (RPS28 and RPS15) to enhance the cell apoptosis process,^102^ and 5′-tRF-Gly directly targets CEACAM1 (carcinoembryonic antigen-related cell adhesion molecule 1) to reduce tumor size and metastasis.^77^

### Biliary tract disease

Research on tsRNA involvement in biliary tract diseases is limited, but studies have reported that tsRNAs can predict overall survival in patients with gallbladder cancer and explore related mechanisms. For instance, Zou et al reported significantly down-regulated tRF-3013b in gallbladder cancer, and it was closely related to the overall survival rate.^78^ Mechanistically, TRF-3013b can inhibit tumor cell proliferation and induce cell cycle arrest by binding to AGO3, which is a target of TPRG1L (tumor protein p63 regulated 1 like).^78^ These findings suggest that tsRNAs may serve as potential therapeutic targets for gallbladder cancer, but more evidence is needed.

### Pancreatic disease

The potential roles of tsRNAs in pancreatic diseases, specifically pancreatic cancer and PDAC, have been extensively investigated by researchers. Jin et al reported 48 differentially expressed tsRNAs in pancreatic cancer samples. Among these tsRNAs, up-regulation of AS-tDR-000064, AS-tDR-000069, and AS-tDR-000102 emphasized their potential as biomarkers for pancreatic cancer.^87^ Another study identified 45 tsRNAs with significantly higher expression levels and 6 tsRNAs with lower expression levels in patients with pancreatic cancer. Furthermore, they evaluated the accuracy of tsRNA-ValTAC-41 and tsRNA-MetCAT-37 in the differential diagnosis of PDAC.^62^ Further studies on a specific tsRNA called tRF-21-VBY9PYKHD (tRF-21) are advancing our understanding of the role of tsRNAs in PDAC and uncovering potential therapeutic applications. In *in vivo* models, down-regulated tRF-21 exerted a tumor-inhibitory effect. However, reduced tRF-21 was observed to promote the malignant phenotype of PDAC cells *in vitro*.^66^ In addition, tRF-19-Q1Q89PJZ inhibits the malignant activity of pancreatic cancer cells by regulating HK1 (hexokinase 1)-mediated glycolysis.^79^ TRF-19-PNR8YPJZ activates the Wnt signaling pathway by targeting AXIN2 (axin 2),^80^ while tRF-Leu-AAG targets UPF1 (Up-frameshift protein 1),^103^ they promote proliferation and metastasis of pancreatic cancer cells. As mentioned above, these findings highlight the potential of tsRNAs as diagnostic markers in PDAC, but more research in this field may lead to the development of improved diagnostic methods and therapeutic strategies for these diseases.

Another study conducted by Yang et al investigated the mechanism of tsRNA involvement in acute pancreatitis via bioinformatics methods. They identified 19 central tRFs associated with pancreatic acinar intracellular trypsinogen activation which is a key event in acute pancreatitis. Specifically, down-regulated tRF3-Thr-AGT may contribute to the pancreatic acinar intracellular trypsinogen activation and the subsequent inflammatory response observed in acute pancreatitis.^104^ Through molecular biology experiments, Fan et al validated that the binding of tRF-36 and IGF2BP3 (insulin-like growth factor 2 mRNA binding protein 3) accelerates the progression of acute pancreatitis.^81^ These studies provide a basis for understanding the regulatory mechanism of tsRNA in acute pancreatitis.

## Conclusion and prospects

According to current perspectives, tsRNAs are increasingly believed to play a crucial role in the occurrence and development of different digestive tract diseases. As small RNA molecules that are generated through precise cleavage, tsRNAs are observed to be widely present and highly conserved in digestive tract diseases. This review highlights the significance of tsRNAs in both clinical and basic research on gastrointestinal diseases and emphasizes the need to consider their potential roles in disease diagnosis, prognosis prediction, and therapeutic strategies.

Two primary potential clinical implications were identified from the studies discussed in this review. On the one hand, certain tsRNAs, such as tRF-38-QB1MC8YUBS68BFD2, tRF-18-BS68BFD1, tRF-25-R9ODMJ6B26, tRF-31-U5YKFN8DYDZDD, tRF-phe-GAA-031, and tRF-VAL-TCA-002, are associated with disease diagnosis and prognosis prediction. Many studies have confirmed the high sensitivity and accuracy of tsRNA in diagnosis and prediction, but it is worth further exploring whether the expression and efficacy of tsRNA from different sources, such as serum, plasma exosomes, or tissues, are consistent. On the other hand, perhaps due to the chronic and recurrent nature of tumor-related diseases, tsRNA has been detected and used for the diagnosis and prediction of more gastrointestinal tumors, demonstrating high sensitivity and accuracy. However, few studies have explored the role of tsRNA in the clinical diagnosis of non-tumor gastrointestinal diseases. Moreover, these tsRNAs participate in digestive tract diseases through different signaling pathways. Most studies have focused on regulating biological functions such as immune response, tumor cell proliferation, differentiation, and apoptosis in tumor-related digestive tract diseases. Studies have reported the effects of tsRNAs on metabolism, inflammatory response, and viral infection in non-tumor-related digestive tract diseases, indicating that these tsRNAs may be potential targets for treating these diseases. Multiple studies have used tsRNA Agomir and Antagonir formulations to simulate or inhibit the expression of tsRNA in *in vivo* models, successfully intervening in disease phenotypes. tsRNA-targeted interventions have the potential for tissue specificity and patient universality. If various chemical modification modes and drug delivery systems are developed based on delivery materials such as lipids, exosomes, and inorganic nanoparticles, efficient tsRNA drugs may be developed for clinical treatment, making tsRNA intervention an effective treatment option. While there has been progress in tsRNA research, many areas still require further exploration. Future research in this field is expected to shed more light on the underlying mechanisms of tsRNAs involved in digestive tract diseases and offer new insights for developing innovative treatment approaches.

## Author contributions

M.L. drafted the initial manuscript. X.Z. and H.Z. checked and revised the manuscript. G.Y. and W.J. participated in the review design and helped modify the manuscript. All authors read and approved the final version of the manuscript.

## Funding

This project was supported by the 10.13039/501100001809National Natural Science Foundation of China (No. 82073047, 82272995) and Guangdong Provincial Basic and Applied Basic Research Foundation Natural Science Foundation (China) (No. 2022A1515010954).

## Conflict of interests

The authors declared no competing interests.

## References

[1] Xie Y., Yao L., Yu X., Ruan Y., Li Z., Guo J. (2020). Action mechanisms and research methods of tRNA-derived small RNAs. Signal Transduct Targeted Ther.

[2] Borek E., Baliga B.S., Gehrke C.W. (1977). High turnover rate of transfer RNA in tumor tissue. Cancer Res.

[3] Wang Q., Lee I., Ren J., Ajay S.S., Lee Y.S., Bao X. (2013). Identification and functional characterization of tRNA-derived RNA fragments (tRFs) in respiratory syncytial virus infection. Mol Ther.

[4] Wang C., Chen W., Aili M., Zhu L., Chen Y. (2023). tRNA-derived small RNAs in plant response to biotic and abiotic stresses. Front Plant Sci.

[5] Peng R., Santos H.J., Nozaki T. (2022). Transfer RNA-derived small RNAs in the pathogenesis of parasitic *Protozoa*. Genes.

[6] Weng Q., Wang Y., Xie Y. (2022). Extracellular vesicles-associated tRNA-derived fragments (tRFs): biogenesis, biological functions, and their role as potential biomarkers in human diseases. J Mol Med.

[7] Anastasiadou E., Jacob L.S., Slack F.J. (2018). Non-coding RNA networks in cancer. Nat Rev Cancer.

[8] Liu B., Cao J., Wang X., Guo C., Liu Y., Wang T. (2021). Deciphering the tRNA-derived small RNAs: origin, development, and future. Cell Death Dis.

[9] Wang Y., Weng Q., Ge J., Zhang X., Guo J., Ye G. (2022). tRNA-derived small RNAs: mechanisms and potential roles in cancers. Genes Dis.

[10] Zhu L., Ge J., Li T., Shen Y., Guo J. (2019). tRNA-derived fragments and tRNA halves: the new players in cancers. Cancer Lett.

[11] Li S., Xu Z., Sheng J. (2018). tRNA-derived small RNA: a novel regulatory small non-coding RNA. Genes.

[12] Kim H.K., Yeom J.H., Kay M.A. (2020). Transfer RNA-derived small RNAs: Another layer of gene regulation and novel targets for disease therapeutics. Mol Ther.

[13] Tao E.W., Cheng W.Y., Li W.L., Yu J., Gao Q.Y. (2020). tiRNAs: a novel class of small noncoding RNAs that helps cells respond to stressors and plays roles in cancer progression. J Cell Physiol.

[14] Jiang P., Yan F. (2019). tiRNAs & tRFs biogenesis and regulation of diseases: a review. Curr Med Chem.

[15] Shen Y., Yu X., Zhu L., Li T., Yan Z., Guo J. (2018). Transfer RNA-derived fragments and tRNA halves: biogenesis, biological functions and their roles in diseases. J Mol Med.

[16] Gebetsberger J., Polacek N. (2013). Slicing tRNAs to boost functional ncRNA diversity. RNA Biol.

[17] Kumar P., Kuscu C., Dutta A. (2016). Biogenesis and function of transfer RNA-related fragments (tRFs). Trends Biochem Sci.

[18] Goodarzi H., Liu X., Nguyen H.C.B., Zhang S., Fish L., Tavazoie S.F. (2015). Endogenous tRNA-derived fragments suppress breast cancer progression via YBX1 displacement. Cell.

[19] Zeng T., Hua Y., Sun C. (2020). Relationship between tRNA-derived fragments and human cancers. Int J Cancer.

[20] Alves C.S., Nogueira F.T.S. (2021). Plant small RNA world growing bigger: tRNA-derived fragments, longstanding players in regulatory processes. Front Mol Biosci.

[21] Zong T., Yang Y., Zhao H. (2021). tsRNAs: novel small molecules from cell function and regulatory mechanism to therapeutic targets. Cell Prolif.

[22] Yamasaki S., Ivanov P., Hu G.F., Anderson P. (2009). Angiogenin cleaves tRNA and promotes stress-induced translational repression. J Cell Biol.

[23] Zhang X., Cozen A.E., Liu Y., Chen Q., Lowe T.M. (2016). Small RNA modifications: integral to function and disease. Trends Mol Med.

[24] Guan L., Karaiskos S., Grigoriev A. (2020). Inferring targeting modes of Argonaute-loaded tRNA fragments. RNA Biol.

[25] Wu J.E., Yang J., Cho W.C., Zheng Y. (2020). Argonaute proteins: structural features, functions and emerging roles. J Adv Res.

[26] Green J.A., Ansari M.Y., Ball H.C., Haqqi T.M. (2020). tRNA-derived fragments (tRFs) regulate post-transcriptional gene expression via AGO-dependent mechanism in IL-1β stimulated chondrocytes. Osteoarthritis Cartilage.

[27] Yeung M.L., Bennasser Y., Watashi K., Le S.Y., Houzet L., Jeang K.T. (2009). Pyrosequencing of small non-coding RNAs in HIV-1 infected cells: evidence for the processing of a viral-cellular double-stranded RNA hybrid. Nucleic Acids Res.

[28] Zhong F., Hu Z., Jiang K. (2019). Complement C3 activation regulates the production of tRNA-derived fragments Gly-tRFs and promotes alcohol-induced liver injury and steatosis. Cell Res.

[29] Lakshmanan V., Sujith T.N., Bansal D., Shivaprasad P.V., Palakodeti D., Krishna S. (2021). Comprehensive annotation and characterization of planarian tRNA and tRNA-derived fragments (tRFs). RNA.

[30] Yang D., Xiao F., Yuan Y. (2023). The expression pattern of tRNA-derived small RNAs in adult *Drosophila* and the function of tRF-Trp-CCA-014-H3C4 network analysis. Int J Mol Sci.

[31] Luo S., He F., Luo J. (2018). *Drosophila* tsRNAs preferentially suppress general translation machinery via antisense pairing and participate in cellular starvation response. Nucleic Acids Res.

[32] Han L., Lai H., Yang Y. (2021). A 5'-tRNA halve, tiRNA-Gly promotes cell proliferation and migration via binding to RBM17 and inducing alternative splicing in papillary thyroid cancer. J Exp Clin Cancer Res.

[33] Krishna S., Yim D.G., Lakshmanan V. (2019). Dynamic expression of tRNA-derived small RNAs define cellular states. EMBO Rep.

[34] Sobala A., Hutvagner G. (2013). Small RNAs derived from the 5' end of tRNA can inhibit protein translation in human cells. RNA Biol.

[35] Yu X., Xie Y., Zhang S., Song X., Xiao B., Yan Z. (2021). tRNA-derived fragments: mechanisms underlying their regulation of gene expression and potential applications as therapeutic targets in cancers and virus infections. Theranostics.

[36] Ivanov P., Emara M.M., Villen J., Gygi S.P., Anderson P. (2011). Angiogenin-induced tRNA fragments inhibit translation initiation. Mol Cell.

[37] Gebetsberger J., Zywicki M., Künzi A., Polacek N. (2012). tRNA-derived fragments target the ribosome and function as regulatory non-coding RNA in *Haloferax volcanii*. Archaea.

[38] Cho H., Lee W., Kim G.W. (2019). Regulation of La/SSB-dependent viral gene expression by pre-tRNA 3' trailer-derived tRNA fragments. Nucleic Acids Res.

[39] Lalande S., Merret R., Salinas-Giegé T., Drouard L. (2020). *Arabidopsis* tRNA-derived fragments as potential modulators of translation. RNA Biol.

[40] Bąkowska-Żywicka K., Kasprzyk M., Twardowski T. (2016). tRNA-derived short RNAs bind to *Saccharomyces cerevisiae* ribosomes in a stress-dependent manner and inhibit protein synthesis *in vitro*. FEMS Yeast Res.

[41] Fricker R., Brogli R., Luidalepp H. (2019). A tRNA half modulates translation as stress response in *Trypanosoma brucei*. Nat Commun.

[42] Watanabe T., Tomizawa S.I., Mitsuya K. (2011). Role for piRNAs and noncoding RNA in *de novo* DNA methylation of the imprinted mouse *Rasgrf1* locus. Science.

[43] Schorn A.J., Gutbrod M.J., LeBlanc C., Martienssen R. (2017). LTR-retrotransposon control by tRNA-derived small RNAs. Cell.

[44] Siomi M.C., Sato K., Pezic D., Aravin A.A. (2011). PIWI-interacting small RNAs: the vanguard of genome defence. Nat Rev Mol Cell Biol.

[45] Boskovic A., Bing X.Y., Kaymak E., Rando O.J. (2020). Control of noncoding RNA production and histone levels by a 5' tRNA fragment. Genes Dev.

[46] Durdevic Z., Mobin M.B., Hanna K., Lyko F., Schaefer M. (2013). The RNA methyltransferase Dnmt2 is required for efficient Dicer-2-dependent siRNA pathway activity in *Drosophila*. Cell Rep.

[47] Park J., Ahn S.H., Shin M.G., Kim H.K., Chang S. (2020). tRNA-derived small RNAs: novel epigenetic regulators. Cancers.

[48] Li X., Zhang Y., Li Y., Gu X., Ju S. (2023). A comprehensive evaluation of serum tRF-29-R9J8909NF_5_JP as a novel diagnostic and prognostic biomarker for gastric cancer. Mol Carcinog.

[49] Shen Y., Xie Y., Yu X. (2021). Clinical diagnostic values of transfer RNA-derived fragment tRF-19-3L7L73JD and its effects on the growth of gastric cancer cells. J Cancer.

[50] Shen Y., Yu X., Ruan Y. (2021). Global profile of tRNA-derived small RNAs in gastric cancer patient plasma and identification of tRF-33-P4R8YP9LON4VDP as a new tumor suppressor. Int J Med Sci.

[51] Zhang Y., Gu X., Qin X., Huang Y., Ju S. (2022). Evaluation of serum tRF-23-Q99P9P9NDD as a potential biomarker for the clinical diagnosis of gastric cancer. Mol Med.

[52] Li Y., Zhang Y., Li X., Li X., Gu X., Ju S. (2023). Serum tRF-27-FDXXE6XRK45 as a promising biomarker for the clinical diagnosis in gastric cancer. Int J Med Sci.

[53] Zhu L., Li T., Shen Y., Yu X., Xiao B., Guo J. (2019). Using tRNA halves as novel biomarkers for the diagnosis of gastric cancer. Cancer Biomarkers.

[54] Lin C., Zheng L., Huang R., Yang G., Chen J., Li H. (2020). tRFs as potential exosome tRNA-derived fragment biomarkers for gastric carcinoma. Clin Lab.

[55] Yu X., Song X., Xie Y., Zhang S., Guo J. (2022). Establishment of an absolute quantitative method to detect a plasma tRNA-derived fragment and its application in the non-invasive diagnosis of gastric cancer. Int J Mol Sci.

[56] Huang Y., Zhang H., Gu X. (2021). Elucidating the role of serum tRF-31-U_5_YKFN8DYDZDD as a novel diagnostic biomarker in gastric cancer (GC). Front Oncol.

[57] Zhu L., Li Z., Yu X. (2021). The tRNA-derived fragment 5026a inhibits the proliferation of gastric cancer cells by regulating the PTEN/PI3K/AKT signaling pathway. Stem Cell Res Ther.

[58] Li S., Shi X., Chen M. (2019). Angiogenin promotes colorectal cancer metastasis *via* tiRNA production. Int J Cancer.

[59] Wu Y., Yang X., Jiang G. (2021). 5'-tRF-GlyGCC: a tRNA-derived small RNA as a novel biomarker for colorectal cancer diagnosis. Genome Med.

[60] Zhu L., Li J., Gong Y. (2019). Exosomal tRNA-derived small RNA as a promising biomarker for cancer diagnosis. Mol Cancer.

[61] Huang P., Tu B., Liao H.J. (2021). Elevation of plasma tRNA fragments as a promising biomarker for liver fibrosis in nonalcoholic fatty liver disease. Sci Rep.

[62] Xue M., Shi M., Xie J. (2021). Serum tRNA-derived small RNAs as potential novel diagnostic biomarkers for pancreatic ductal adenocarcinoma. Am J Cancer Res.

[63] Panoutsopoulou K., Dreyer T., Dorn J. (2021). tRNAGlyGCC-derived internal fragment (i-tRF-GlyGCC) in ovarian cancer treatment outcome and progression. Cancers.

[64] Tsiakanikas P., Adamopoulos P.G., Tsirba D. (2022). High expression of a tRNAPro derivative associates with poor survival and independently predicts colorectal cancer recurrence. Biomedicines.

[65] Zhu Y., Chen S., Ling Z. (2021). Comprehensive analysis of a tRNA-derived small RNA in colorectal cancer. Front Oncol.

[66] Pan L., Huang X., Liu Z.X. (2021). Inflammatory cytokine-regulated tRNA-derived fragment tRF-21 suppresses pancreatic ductal adenocarcinoma progression. J Clin Investig.

[67] Gu X., Ma S., Liang B., Ju S. (2021). Serum hsa_tsr016141 as a kind of tRNA-derived fragments is a novel biomarker in gastric cancer. Front Oncol.

[68] Cui H., Li H., Wu H. (2022). A novel 3'tRNA-derived fragment tRF-Val promotes proliferation and inhibits apoptosis by targeting EEF1A1 in gastric cancer. Cell Death Dis.

[69] Zheng J., Li C., Zhu Z. (2022). A 5`-tRNA derived fragment NamedtiRNA-Val-CAC-001 works as a suppressor in gastric cancer. Cancer Manag Res.

[70] Wang Y., Li Z., Weng Q. (2023). Clinical diagnostic values of transfer RNA-derived fragment tRF-41-YDLBRY_73_W_0_K_5_KKOVD and its effects on the growth of gastric cancer cells. DNA Cell Biol.

[71] Zhang F., Shi J., Wu Z. (2020). A 3'-tRNA-derived fragment enhances cell proliferation, migration and invasion in gastric cancer by targeting FBXO47. Arch Biochem Biophys.

[72] Tao E.W., Wang H.L., Cheng W.Y., Liu Q.Q., Chen Y.X., Gao Q.Y. (2021). A specific tRNA half, 5'tiRNA-His-GTG, responds to hypoxia via the HIF1α/ANG axis and promotes colorectal cancer progression by regulating LATS2. J Exp Clin Cancer Res.

[73] Huang T., Chen C., Du J. (2023). A tRF-5a fragment that regulates radiation resistance of colorectal cancer cells by targeting *MKNK1*. J Cell Mol Med.

[74] Luan N., Wang J., Sheng B. (2023). tRF-20-M0NK5Y93-induced MALAT1 promotes colon cancer metastasis through alternative splicing of SMC1A. Am J Cancer Res.

[75] Zhou Y., Hu J., Liu L. (2021). Gly-tRF enhances LCSC-like properties and promotes HCC cells migration by targeting NDFIP2. Cancer Cell Int.

[76] Zhu J., Cheng M., Zhao X. (2020). A tRNA-derived fragment (tRF-3001b) aggravates the development of nonalcoholic fatty liver disease by inhibiting autophagy. Life Sci.

[77] Liu D., Wu C., Wang J. (2022). Transfer RNA-derived fragment 5'tRF-Gly promotes the development of hepatocellular carcinoma by direct targeting of carcinoembryonic antigen-related cell adhesion molecule 1. Cancer Sci.

[78] Zou L., Yang Y., Zhou B. (2022). tRF-3013b inhibits gallbladder cancer proliferation by targeting TPRG1L. Cell Mol Biol Lett.

[79] Cao W., Zeng Z., Lei S. (2023). 5’-tRF-19-Q1Q89PJZ suppresses the proliferation and metastasis of pancreatic cancer cells via regulating hexokinase 1-mediated glycolysis. Biomolecules.

[80] Cao W., Dai S., Ruan W., Long T., Zeng Z., Lei S. (2023). Pancreatic stellate cell-derived exosomal tRF-19-PNR8YPJZ promotes proliferation and mobility of pancreatic cancer through AXIN_2_. J Cell Mol Med.

[81] Fan X.R., Huang Y., Su Y. (2023). Exploring the regulatory mechanism of tRNA-derived fragments 36 in acute pancreatitis based on small RNA sequencing and experiments. World J Gastroenterol.

[82] Xu W., Zhou B., Wang J. (2021). tRNA-derived fragment tRF-Glu-TTC-027 regulates the progression of gastric carcinoma *via* MAPK signaling pathway. Front Oncol.

[83] Xu W., Zheng J., Wang X. (2022). tRF-Val-CAC-016 modulates the transduction of CACNA1d-mediated MAPK signaling pathways to suppress the proliferation of gastric carcinoma. Cell Commun Signal.

[84] Dong X., Fan X., He X. (2020). Comprehensively identifying the key tRNA-derived fragments and investigating their function in gastric cancer processes. OncoTargets Ther.

[85] Huang B., Yang H., Cheng X. (2017). tRF/miR-1280 suppresses stem cell-like cells and metastasis in colorectal cancer. Cancer Res.

[86] Wang B., Xia L., Zhu D. (2022). Paternal high-fat diet altered sperm 5'tsRNA-gly-GCC is associated with enhanced gluconeogenesis in the offspring. Front Mol Biosci.

[87] Jin L., Zhu C., Qin X. (2019). Expression profile of tRNA-derived fragments in pancreatic cancer. Oncol Lett.

[88] Tong L., Zhang W., Qu B. (2021). The tRNA-derived fragment-3017A promotes metastasis by inhibiting NELL2 in human gastric cancer. Front Oncol.

[89] Wang X., Zhang Y., Ghareeb W.M. (2020). A comprehensive repertoire of transfer RNA-derived fragments and their regulatory networks in colorectal cancer. J Comput Biol.

[90] Chen H., Xu Z., Cai H., Peng Y., Yang L., Wang Z. (2022). Identifying differentially expressed tRNA-derived small fragments as a biomarker for the progression and metastasis of colorectal cancer. Dis Markers.

[91] Han Y., Peng Y., Liu S. (2022). tRF3008A suppresses the progression and metastasis of colorectal cancer by destabilizing FOXK1 in an AGO-dependent manner. J Exp Clin Cancer Res.

[92] Lu S., Wei X., Tao L. (2022). A novel tRNA-derived fragment tRF-3022b modulates cell apoptosis and M2 macrophage polarization via binding to cytokines in colorectal cancer. J Hematol Oncol.

[93] Luan N., Mu Y., Mu J. (2021). Dicer1 promotes colon cancer cell invasion and migration through modulation of tRF-20-MEJB5Y13 expression under hypoxia. Front Genet.

[94] Xiong W., Wang X., Cai X. (2019). Identification of tRNA-derived fragments in colon cancer by comprehensive small RNA sequencing. Oncol Rep.

[95] Yang C., Song J., Park S. (2022). Targeting thymidylate synthase and tRNA-derived non-coding RNAs improves therapeutic sensitivity in colorectal cancer. Antioxidants.

[96] Cao K.Y., Pan Y., Yan T.M., Tao P., Xiao Y., Jiang Z.H. (2022). Antitumor activities of tRNA-derived fragments and tRNA halves from non-pathogenic *Escherichia coli* strains on colorectal cancer and their structure-activity relationship. mSystems.

[97] Ying S., Li P., Wang J. (2023). tRF-Gln-CTG-026 ameliorates liver injury by alleviating global protein synthesis. Signal Transduct Targeted Ther.

[98] Tzur Y., Winek K., Madrer N. (2024). Lysine tRNA fragments and miR-194-5p co-regulate hepatic steatosis via β-Klotho and perilipin 2. Mol Metabol.

[99] Zhu J., Wen Y., Zhang Q., Nie F., Cheng M., Zhao X. (2022). The monomer TEC of blueberry improves NASH by augmenting tRF-47-mediated autophagy/pyroptosis signaling pathway. J Transl Med.

[100] Zhan S., Yang P., Zhou S. (2022). Serum mitochondrial tsRNA serves as a novel biomarker for hepatocarcinoma diagnosis. Front Med.

[101] Wu C., Liu D., Zhang L. (2023). 5'-tiRNA-Gln inhibits hepatocellular carcinoma progression by repressing translation through the interaction with eukaryotic initiation factor 4A-I. Front Med.

[102] Kim H.K., Fuchs G., Wang S. (2017). A transfer-RNA-derived small RNA regulates ribosome biogenesis. Nature.

[103] Sui S., Wang Z., Cui X., Jin L., Zhu C. (2022). The biological behavior of tRNA-derived fragment tRF-Leu-AAG in pancreatic cancer cells. Bioengineered.

[104] Yang H., Zhang H., Chen Z., Wang Y., Gao B. (2022). Effects of tRNA-derived fragments and microRNAs regulatory network on pancreatic acinar intracellular trypsinogen activation. Bioengineered.

